# *“We are only looking at the tip of the iceberg in infertility”*: perspectives of health providers about fertility issues and management among Aboriginal and Torres Strait Islander people

**DOI:** 10.1186/s12913-021-06714-8

**Published:** 2021-07-17

**Authors:** Emily Gilbert, Ruth Walker, David Simon, Ruth Derkenne, Alice Rumbold, Sandra Campbell, Jacqueline A Boyle

**Affiliations:** 1 Monash Centre for Health Research and Implementation, School of Public Health and Preventative Medicine, Monash University, VIC Melbourne, Australia; 2 Top End Health Service, NT Casuarina, Australia; 3 South Australian Health and Medical Research Institute, SA Adelaide, Australia; 4 Molly Wardaguga Research Centre, School of Nursing and Midwifery, Charles Darwin University, QLD Brisbane City, Australia

**Keywords:** Aboriginal and Torres Strait Islander, fertility, Indigenous, infertility, reproductive health, assisted reproductive technology, IVF

## Abstract

**Introduction:**

Aboriginal and Torres Strait Islander women and men are disproportionately affected by a range of risk factors for infertility. However, remarkably little is known about the prevalence of infertility in this group, or how Aboriginal and Torres Strait Islander people access fertility treatments including assisted reproductive technology (ART). This qualitative study aims to explore health care provider (HCP) perspectives on the health burden of infertility among Aboriginal and Torres Strait Islander people, as well as factors that may affect access to infertility treatment for this group.

**Method:**

Semi-structured interviews were conducted with HCPs (8 doctors; 3 nurses and 1 Aboriginal Health Practitioner) working in fertility care in the Northern Territory, Australia. Transcribed interviews were analysed using an iterative thematic approach using the NVivo-9 software package.

**Results:**

Providers perceive infertility as an underestimated health issue in this patient population, reporting a high prevalence of infertility-related risk factors but fewer clinical encounters of diagnosis and treatment. Perceived barriers to accessing fertility care included cultural differences such as the shame and stigma associated with reproductive health and the separation of men’s business and women’s business; service-related barriers such as limited timely and affordable access to specialist health services and; a lack of culturally responsive and appropriate fertility services. Providers had mixed opinions on their role in ameliorating inequities of access, and hence a range of strategies to address barriers were suggested. These included a greater patient education, ongoing patient support and providing a culturally safe environment.

**Conclusion:**

The current study adds to the understanding of how Aboriginal and Torres Strait Islander people access fertility treatments. There is a need for further research to quantify infertility in Aboriginal and Torres Strait Islander people, investigate community perceptions towards infertility and identify community-driven priorities to improve access to fertility care for this population.

**Supplementary Information:**

The online version contains supplementary material available at 10.1186/s12913-021-06714-8.

## Introduction

Infertility affects approximately 15 % of reproductive-aged couples, causing significant social and psychological problems [1]. Assisted reproductive technologies (ART), such as IVF (in vitro fertilisation) can be used to overcome a range of fertility issues. Globally, the use of ART has steadily increased over the last three decades [2]. The latest estimates indicate that over 1.6 million ART cycles are reported worldwide each year, with 400,000 babies being born [3]. Increased ART utilisation is being driven by a multitude of factors such as the rise of infertility owing to the impact of lifestyle factors (e.g. delayed parenthood, obesity, psychological stress etc.), [4] improved accessibility in terms the number and location of clinics, and the availability of low-cost fertility options [5]. Despite ART use increasing globally, widespread disparities in access to treatment exist, both between and within countries [2]. Across Africa, 33 out of 54 countries (61 %) have no registered IVF unit, [6] despite elevated levels of secondary infertility in most countries [7]. This contrasts with Europe where nearly half (42.7 %) of all the world’s fertility centres are based [2]. In the United States (US), African American, Hispanics, and persons of middle to lower socio-economic status are underrepresented in the population of infertility patients [8]. Moreover, among women who do seek treatment to become pregnant, African American women usually have a longer duration of infertility problems before seeking care than their white counterparts, potentially contributing to lower success rates [9]. In Australia, data from the Australian and New Zealand Assisted Reproduction Database (ANZARD)^1^ report shows socioeconomic and geographical disparities in access to ART, even after adjusting for need [10]. Barriers associated with treatment costs occur in part because although treatment is partially funded by the Commonwealth Government under the Medicare Benefits Scheme, [11] most treatments require co-payments, and costs vary across clinics and jurisdictions [12, 13]. For some treatments, out of pocket costs can be substantial (e.g. estimated cost of fertility provider IVF Australia for one IVF cycle is $4484 [13]), and certain items are not covered by Medicare at all (e.g. hospital/day surgery related services). To reduce out of pocket costs, some services offer bulk-billed IVF services, lower cost treatment strategies and discounts to concession card holders, however, it is unclear how widespread these practices are and even with these measures the cost can remain prohibitive for some.

Currently ANZARD does not collect ethnicity data or Indigenous status, therefore at a national level disparity in access to ART between different ethnic groups remains unknown. To our knowledge there are no published studies at community-level.

In Australia, Aboriginal and Torres Strait Islander people experience greater socioeconomic disadvantage than their non-Indigenous counterparts, a reflection of the ongoing impact of colonisation [14]. This places Aboriginal and Torres Strait Islander people at greater risk of exposure to behavioural and environmental health risk factors, including several of the most influential factors on fertility namely tobacco smoking, risky alcohol consumption, physical inactivity, obesity and sexually transmitted infections (STIs) [15]. Conditions that can adversely affect fertility such as polycystic ovary syndrome (PCOS), metabolic syndrome and diabetes are also more common [15, 16] and there is some evidence, albeit very limited, that suggest the rate of infertility, especially secondary infertility, in this group is high [17].

Despite evidence of elevated risk of infertility, national figures show a higher total birth rate and a younger median age of giving birth for Aboriginal and Torres Strait Islander women compared to all Australian women (2.3 births per woman compared with 1.9 per woman) [14]. This incongruence between suspected and actual prevalence of infertility for Aboriginal and Torres Strait Islander people raises concerns that the relatively high birth rate could mask significant rates of infertility, with those affected remaining largely undiagnosed and untreated [18]. This may in part reflect the fact Aboriginal and Torres Strait Islander people have poorer access to and use of mainstream health services, due to barriers such as geographic isolation, the high cost of health care, culture and language differences, and experiences of racism and discrimination [19]. Additionally, access to infertility care may be further complicated by barriers specific to infertility treatment, including community perceptions of infertility and infertility care, high out-of-pocket costs, insufficient knowledge about the IVF process, and feelings of shame and isolation [20, 21].

Multiple factors potentially affect access to infertility care and ART for Aboriginal and Torres Strait Islander people, however to date, no studies have explicitly investigated these factors. As treating providers, health care providers (HCPs) have unique insight into where barriers to care potentially exist. Therefore, the aim of this study was to qualitatively explore the perceptions of HCPs on the  burden of infertility among Aboriginal and Torres Strait Islander people, as well as the barriers and facilitators to infertility treatment for this group.

## Methods

### Ethics

Ethics approval for this project was obtained from the Human Research Ethics Committee of Northern Territory Department of Health and Menzies School of Health Research (HREC reference: 2019–3432).

### Design

Taking a descriptive qualitative approach, semi-structured in-depth interviews were conducted with HCPs working in fertility care and/or sexual and reproductive health care in Darwin and remote communities across the Top End of Northern Territory (NT), Australia, from November to December 2019. The planning and reporting of this research was guided by the Consolidated Criteria for Reporting Qualitative Research [22].

### Study setting

The NT is situated in northern-central Australia, covering an area of 1.42 million km^2^. The NT has a markedly different population profile, compared to other jurisdictions in Australia. The NT’s population is younger, more sparsely populated and highly transient, posing significant challenges to healthcare delivery. Aboriginal and Torres Strait Islander people make up approximately 30 % of the population (compared with 5.5 % or less for all other jurisdictions), of which a high proportion live in rural and remote areas. Rates of socioeconomic disadvantage are high, particularly among Aboriginal and Torres Strait Islander people. Health care in remote communities is provided by a mix of clinicians including general practitioners (primary healthcare physicians), nurses and midwives who may be resident in the community or visit, Aboriginal Health Practitioners who are resident members of the community in possession of a minimum qualification (certificate III) within the fields of primary health care work or clinical practice and specialists such as gynaecologists who may visit every few months. There are usually no pathology or radiology services. There is one ART unit based in the capital city largely managed by a small team of clinicians (general practitioners and nursing staff) with additional support from visiting specialists (gynaecologists) based in another state.

### Recruitment

Participants were recruited using purposive and snowball sampling. Members of the research team identified a range of providers through their clinical networks who were likely to be information rich and give a variety of insights based on age, gender, professional roles and experience. Participants were asked to nominate other relevant HCPs to be interviewed. Prospective participants were contacted were provided with an information sheet and consent form via email.

### Data collection

The primary researcher (EG) conducted semi-structured interviews face-to-face, and alternatively via telephone. In instances where face-to-face mode was not possible, reasons included issues of cost and travel and time constraints of HCPs. At the time of data collection, EG was a PhD student (trained to a MClin Embryol. in Medical research) who had been involved in prior qualitative research studies. Interviews comprised 10 open-ended questions – based on an interview guide developed by the research team of clinicians and researchers working in the field of fertility care and/or Aboriginal and Torres Strait Islander health. Questions examined demographic information and the HCP’s perception of (1) burden of infertility; (2) access and uptake of fertility services; and (3) barriers and facilitators to care, in relation to Aboriginal and Torres Strait Islander people (supplementary material 1). Sample size requirement was informed by previous research where up to 15 interviews enabled broad coverage and data saturation [23].

### Data analysis

Interviews were audio recorded, transcribed verbatim by the primary researcher (EG), and entered into the NVivo 9 software package for data analysis. Iterative thematic analysis was undertaken using the six-step framework described by Braun and Clarke [24]. An inductive process was chosen to analyse the data. Two authors (EG, RW), one who had been involved in prior qualitative research studies (EG), and the other a clinician-researcher with extensive experience in qualitative methods (RW), read two randomly selected transcripts several times to familiarise themselves with the data before establishing an initial coding framework. The same two authors then coded all transcripts separately, meeting regularly to discuss any new codes or differences in coding. When differences arose, the coders reviewed the transcripts again until consensus was achieved. Codes were grouped into categories according to their similarities and similar categories were combined into overarching themes.

## Results

### Participants

Twelve HCPs (8 doctors, 3 nurses and 1 Aboriginal Health Practitioner) were interviewed. Interviews were conducted in person (*n* = 6) or by telephone (*n* = 6), with a mean duration of 40 min (range 25 to 59 min). Respondents were mostly female (*n* = 8) and aged between 50 and 59 years (*n* = 5). Duration of employment in the NT varied, with only one respondent born in the NT. The majority (*n* = 11) of participants currently worked predominately in the public health sector. Most of the respondents reported having worked in a mix of metropolitan, rural or remote health medicine. Approximately half currently lived and worked in remote areas. Of the 8 doctors, 5 had specialist training in General Practice or Rural Generalism, and the others in Obstetrics and Gynaecology. Two participants worked in the ART clinic.

### Themes

Three overarching themes were identified: (i) experiences of infertility in the community; (ii) challenges in providing infertility care; and (iii) navigating the issue of infertility. Subthemes within each theme were identified to accurately and clearly represent the data.

#### Theme 1: experiences of infertility in the community

Providers suspect infertility to be an issue of concern in Aboriginal and Torres Strait Islander communities, despite the lack of empirical evidence to prove it. Underlying reasons included the prevalence of infertility related risk factors, the fact that Aboriginal and Torres Strait Islander peoples’ health issues are often under-estimated and overlooked and perceptions of inequities in access and usage of fertility care in this population.

##### Infertility related risk factors

All HCPs discussed the relatively high rates of infertility-related risk factors in many Aboriginal and Torres Strait Islander communities, compared with the general population. Examples given included recurrent episodes of pelvic inflammatory disease (PID), higher rates of obesity, higher rates of diabetes and higher smoking rates among others. Despite potentially being at higher risk for infertility, HCPs noted infertility was rarely raised as a clinical concern by members of this population group, and consequently referrals to the outreach obstetrician/gynaecologist or specialist fertility clinic were rare occurrences. Providers discussed the issue of secondary infertility with parous women re-partnering and experiencing fertility issues. The frequent occurrence of undiagnosed PID was raised as a likely cause by most HCPs, along with obesity, smoking, diabetes and PCOS. Three providers also noted the higher rates of marijuana smoking in communities and voiced concern about the potential impact on male fertility.

*“What I have seen is a lot of women not on contraception, but not getting pregnant while in relationships. It is infertility but not diagnosed. Then, we have all these women who have expired Implanon who are not getting pregnant, so I think secondary infertility if you started to quantify you would find high rates, but as far as people presenting with primary or secondary it is not that high”.* (HCP, 7: Doctor)

##### Under-estimated and overlooked

Providers raised the possibility of infertility being overlooked by HCPs working with Aboriginal and Torres Strait Islander people. Some suggested infertility was deemed a low-priority care task relative to the chronic and complex health conditions many of their patients face. Providers said whilst patient advocacy was an essential aspect of medical practice, they also have a duty of care and need to consider the impact of providing fertility care to women at increased risk of pregnancy complications.

*“I would say the other issue is, we are so busy dealing with diabetes and other big diseases, that we do not get on to fertility. In the same way we have very low rates of foetal alcohol syndrome screening, and for 50-year-old Indigenous people we are too busy treating COPD, which I am sure means we miss other things and that we only get to cover the top handful of conditions on the problem list”.* (HCP, 9: Doctor)

##### Experiences with care

All HCPs perceived inequities in access to fertility care between Aboriginal and Torres Strait Islander and other Australians and reported infrequent and irregular experiences of infertility care with Aboriginal and Torres Strait Islander populations. An exception was those who worked primarily within the public health system in remote areas and who treated Aboriginal and Torres Strait Islander patients almost exclusively. However, providers working at a specialist fertility clinic acknowledged Aboriginal and Torres Strait Islander status is not routinely collected, which therefore may under-estimate the true proportion seeking care.

“Well, I had one couple from *remote community*, they went through Repromed (IVF Clinic) and we got a pregnancy and that was about 2–3 years ago…There was another lady 8–9 years ago”. (HCP, 5: Doctor)

Provider perception of the duration of infertility prior to investigation varied. One provider spoke about women seeking advice with less than six months of trying and thought that those women probably had a limited understanding of ovulation and the fertile window.

*“It must have happened a couple times as it sticks in my mind, people bring this up [trying to conceive] even less than 6 months of trying to fall pregnant – and this would be young women”* (HCP, 9: Doctor).

Other providers thought it was more likely that Aboriginal and Torres Strait Islander people would wait longer (relative to non-Indigenous people) before raising infertility concerns, due to the significant barriers they are likely to experience and a reluctance among some clients to discuss sexual and reproductive health.

*“I just don’t think Aboriginal and Torres Strait Islander people think that is an option for them because it’s just too much humbug”.* (HCP, 4: AHP)

A few providers felt it was too hard to tell how long women had been trying to get pregnant, explaining that medical records don’t always reflect the entire health history of a patient, and language barriers and cultural misunderstandings make it difficult to obtain missing information.

*“It is hard to tell, the histories that we get, how long women have been trying… a week, a month? How often they have sex, with who, what sort of contraception have they been on etc. - it is all often really vague, so trying to guess that stuff is pretty tricky, but I would have a guess at least a couple of years. With my work as a GP out there talking to women who come through, it is a bit of a taboo topic for a lot of women, and so they won’t mention until it has been at least a few years”.* (HCP, 6: Doctor)

#### Theme 2: Challenges in providing infertility care

Providers perceived a range of barriers to infertility care for Aboriginal and Torres Strait Islander people. The most frequently reported perceived barriers are grouped into cultural, structural and service-related barriers.

##### Cultural barriers

Cultural barriers were discussed at length by all HCPs. Communication and language were widely reported to impede access to and delivery of fertility care. Providers made no mention of using interpreter services, however two HCPs spoke of instances where family members were used as interpreters. Several noted the need for interpreter services within the fertility care system but had concerns regarding whether the particularly complex nature of fertility would translate accurately into language and the shortage of trained medical interpreters. Providers felt these issues were further compounded by a lack of fertility health literacy, particularly in rural and remote communities where cultural and linguistic differences are considered the greatest.

*“We just come along and think just because it’s the way that we think, everyone else thinks that way as well - which is not entirely true. You can talk to someone and think that they understand what you are talking about because they are nodding and saying yes, often because it is easier for them than saying I don’t get any of it… Even if you are using interpreter services - it can become really complicated, because sometimes what is actually written and what it is being interpreted into is two completely different things”. (HCP, 5: Doctor)*

The issue of shame and stigma around fertility-related topics such as not being able to conceive, and sexual health was also raised by several providers as a significant barrier, particularly for men. All providers spoke of women being far more likely than men to raise concerns of infertility and be more actively engaged in fertility care. One provider felt confident that they could recall all their male infertility cases on one hand.

*“There is deep shame some women feel, even for women talking to women, about how often you are having sex, and so it is really difficult for a young woman to have that conversation with stranger.”* (HCP, 3: Nurse).

*“There is [issues with] money, but there is also relationships in that it is quite hard for a lot of women to involve their partners in the process culturally, especially if they have to go off island to do the [fertility] examinations. We have had a lot of issues with getting (semen) samples, which makes the whole process very hard.”* (HCP, 7: Doctor).

The concept of ‘Men’s Business’ and ‘Women’s Business’ recurred throughout the interviews. Participants were confident that many of their patients prefer to speak to someone of the same sex. This is a notable challenge with workforce shortages in rural and remote communities. Most male HCPs expressed concern over the “cultural appropriateness” of engaging in discussions about sexual health with women.

“*I still think there is a cultural barrier which comes from an older male, white male talking to a young Aboriginal girl about sex. What right do I have to do that, apart from my concern for them, and how is that perceived?” (HCP, 8: Doctor)*.

All providers perceived the gendered view of Aboriginal and Torres Strait Islander health, with its separation of “men’s business” and “women’s business”^2^, to be an influence on how this population engage with fertility care. Several providers talked about the likely feelings of shame and embarrassment associated with infertility for many Aboriginal and Torres Strait Islander people, with different implications for men and women. It was commonly reported that typically women come alone, or with female family members, rather than attending as a couple.

Limited male engagement to address fertility issues was perceived as a major barrier in the assessment of the infertile couple.

*“I’ve never seen a couple together, because it is seen as women’s business, and that kind of leads to the problem of getting semen analysis”.* (HCP, 1: Doctor)

*“It is a really big challenge to get the men involved, you can talk to the female partner about smoking and drinking and men’s health as well, but how much that gets relayed to the male partners we can’t know. We don’t see them in gynae-clinic hardly ever”.* (HCP, 6: Doctor)

##### Structural barriers

Overall, providers felt that fertility care services in the NT were inadequate and available services not necessarily appropriate or accessible for Aboriginal and Torres Strait Islander communities, particularly for those living remotely. Practical barriers raised included the distance, time, inconvenience and cost accessing to the nearest health service or specialist fertility clinic. Providers’ also perceived the requirement to travel added stressors such as being away from Country and the emotional support provided by family and community. Providers spoke of how outreach delivery of specialist services have overcome some of the barriers relating to distance. However, they also mentioned challenges relating to the coordination and continuity of care and sequencing of visits which they felt particularly problematic given the complex, time-sensitive nature of fertility care.

*“How the health service and system run, is a massive one (barrier). They see a doctor for a battery of tests, and then no-one comes back to the community for another 6 months. By the time the outreach service is due to come back, it is a different doctor. Someone has to find the results, and if they can’t it is just “oh well we will have to do the tests again”. It almost impossible.”* (HCP, 12:Nurse).

Providers also identified eligibility criteria for assistance with the costs of travel and accommodation for the investigation and treatment of primary and secondary infertility as a barrier. Another barrier proposed was lack of provider knowledge of local infertility services and consequent lack of comfort inquiring or discussing this issue.

“*I don’t have a great understanding of IVF, and what I do know is probably from my wife telling me about friends of hers who have gone through it. Even from medical training, I’ve worked in public hospitals and OB/GYN departments and my understanding is limited, IVF is really is within the domain of private medicine. I’ve never worked in private and therefore as it is largely part of the private health system, I just haven’t had much to do with it. So that actual practical, how many appointments is it going to take, what that are the medications, and the procedures, I couldn’t actually counsel someone one the details which I think would be actually quite important for someone. If I could do that, perhaps it would help someone decide if they want to spend the money?” (HCP, 9: Doctor)*.

##### Service-related barriers

There was consensus among all providers that there is a lack of culturally responsive and appropriate fertility services for the Aboriginal and Torres Strait Islander population. Despite a commitment to providing culturally sensitive care and best efforts with interpersonal interactions, providers said they often felt limited by systemic and organizational constraints.

“There aren’t culturally appropriate services. In order to access any form of reproductive services you have to buy into a total alien system, and that is a lot to ask from people. It is not just the fact you have to come in to Darwin, it is not just the fact that *fertility service* has no male workers, or the difficulty in getting people to understand the whole process, and it is not just the cost. If you had a culturally appropriate service, you would probably get more people who were prepared to look at the cost.” (HCP, 5: Docotr).

A lack of resources was perceived as a significant factor impacting on access to fertility services. Concerns of staff shortages were widespread, particularly among providers working in rural and remote areas, as were issues of skill-mix, scheduling and continuity of care.

*“Interestingly the referrals that come in from remote communities have really dropped in the last year or two., and I think that has a lot to do with the high turnover of staff. I think a lot of the [visiting] gynaecologists that go to remote have a limited understanding of what services are available or how to access them and despite my best efforts that remains a very big barrier”.* (HCP, 11: Doctor)

Workforce shortages placed pressure on HCPs who must manage large patient volumes and increased waiting times for patients. Several providers identified aneed for more Aboriginal and Torres Strait Islander Health Workers and Health Practitioners^3^, however, concerns were raised about employing local community members and maintaining patient confidentiality.

*“People think that we have Aboriginal Health Practitioners (AHPs) out there everywhere, but that is not the case in most communities, is actually really difficult. The other thing is women do not generally want people from their community to help, even if they are AHPs to come in and know their business”.* (HCP, 10: Nurse)

Providers working in smaller regional hospitals and community health clinics spoke of inadequate facilities to deliver infertility diagnoses and treatment remotely. Many couples living in communities seeking infertility care rely on visiting specialist outreach services, however, providers felt the infrequency of outreach visiting makes it difficult to build rapport and maintain trust with the community. Providers reported that scheduled primary care outreach visits seem to rarely correspond to the optimal stage of a woman’s menstrual cycle for time sensitive fertility investigations such as hormone testing. Alternatively, those seeking treatment would travel to regional areas to access hospitals with fertility referral services.

*“If I have a female registrar then that is great, but the last 3 or 4 visits I have been solo which means for the females out there that might not want to talk about their period cycle to me, that limits their access. The remote thing is huge, any tests, whether that is just a simple ultrasound, or a semen analysis or anything high tech that requires flying in and out, most of that is covered by the PATS (Patient Assisted Travel Scheme)*^4^, *but they have kids to look after, and quite often a job out on the island and so you can’t just jump on a plane at the drop of a hat to have a 20 minute ultrasound - so the remote access is a major issue.”* (HCP, 2: Doctor).

Providers felt that because most Aboriginal and Torres Strait Islander people in the NT do not have private health insurance, IVF, with its high cost, was unaffordable for many people.

*“There is a subsidised NT government scheme for Health Care Card holders*^5^ *which allows patients to an IVF cycle for $550 per cycle, which is the total out of pocket treatment cost, except the prescription medicines. However, I think that for many people, of any age, that cost is still a big barrier even though it’s an extremely good deal.”* (HCP, 11: Doctor).

Several providers acknowledged they were unsure of the Medicare rebates in relation to IVF or whether more accessible payment options were offered by fertility clinics. There was some concern that the strict eligibility criteria for PATS assistance meant that the costs of travel and accommodation associated with receiving treatment for infertility was a barrier for many people. Some providers indicated that a clearer understanding of the costs and rebates may change their referral patterns and contribute to better clinical care.

#### Theme 3: Navigating the issue

A general response among providers was that infertility and access to care in Aboriginal and Torres Strait Islander populations, particularly for those living in rural and remote communities, raises distinct and complex challenges. In discussing potential strategies to address barriers identified in Theme 2, there was some concern expressed among HCPs about the potential of opening Pandora’s Box, and differing opinions about the role of the individual provider and the responsibility of fertility services.

##### Pandora’s Box

Some providers felt powerless and poorly equipped to deal with issues of infertility in Aboriginal and Torres Strait Islander populations and expressed concern that in broaching the subject they were ‘opening Pandora’s box’. Providers questioned the ethics of subjecting the social and economic stresses imposed by infertility investigations and treatments on an already vulnerable population group. Diverting resources from other parts of an already stretched health system was perceived as a serious potential problem. Some felt advocacy for this issue should be delayed until provider and services were better positioned to deliver fertility care. In contrast, others felt that HCPs have a duty to provide patients with adequate information make informed decisions about their healthcare.

*“I think anecdotally, we are only looking at the tip of the iceberg in infertility in the community, but we don’t really want to look at it, because we don’t have the resources to do anything about it”.* (HCP, 7: Doctor)

*“If we take away resources to do that (fertility treatment), are we actually taking the resources away from something else? We know from the 1–2 Indigenous people who have gone through treatment that they take an enormous amount of clinical time”.* (HCP, 7: Doctor)

*“I’m really at a dead end here, I’ve put some effort into this year. I don’t think I have been able to penetrate many of these barriers. I probably don’t know what else I can do?”* (HCP, 11: Doctor).

##### Role of the health provider

When providers were asked about their role in removing barriers to access, responses varied. Providers generally felt they had a responsibility to educate patients about fertility-related topics, and many expressed the importance of this beginning in youth and continuing throughout adulthood. It was noted that patient education needs to be comprehensive and easily understood, and that providers have a professional responsibility to keep their knowledge of the latest fertility treatments up to date. Others felt that beyond education, their role was to support patients to navigate their way through the treatment process. It was noted that this often requires providers to “go the extra mile”.

*“I think trying to take the time to spend a bit more time with women to work out how they want to go. Education on the processes and the steps, and what options are available, and explaining exactly what that might look like or that might mean. Being an advocate for my patients, even if that is something that is really hard, if it is something that they really want to pursue, then try to work out a way that we can make it happen, even if it is calling in favours, or going outside of the rules a little bi*t”. (HCP, 6: Doctor)

##### Role of the fertility service

All providers indicated fertility services need to provide culturally safe spaces and appropriate staff. This includes making environments more inviting and respectful of culture, employing Aboriginal people and both male and female staff. The potential benefits of Aboriginal Health Workers, Aboriginal Liaison Officers and interpreter services was also noted by several providers. Some providers felt that fertility services should focus on offering education sessions to primary care HCPs to improve knowledge in fertility treatment options, fees and rebates and referral pathways. Training in cultural competence and accurate collection of data (e.g. Indigenous status on admission) were also acknowledged as important.

Providing public education and resources to raise fertility awareness in community, as well as strengthening community workforce capacity, breaking down stigma and encouraging help seeking were also suggested, with specific efforts to engage men.

*“Being able to offer patients information in a way that they will be able to understand is probably the most important thing, but also providing information not only on fertility services and treatment options but also how the Top End Health Service can assist with the care pathway in terms of PATs and accommodation etc.”* (HCP, 2: Doctor).

## Discussion

To our knowledge, this is the first study to explore HCP perceptions on factors that may affect access to infertility care for Aboriginal and Torres Strait Islander people. Provider’s suspect the prevalence of infertility among Aboriginal and Torres Strait Islander people is high, however, report Aboriginal and Torres Strait Islander people rarely raise concerns of infertility in the clinic and are under-represented in fertility health services. Issues identified by HCPs were grouped into three overlapping categories related to culture, healthcare systems and the provision of services. These factors draw attention to the complexities involved in navigating the issue of infertility in this population group.

Infertility in Aboriginal and Torres Strait Islander people may be a hidden issue. Although national data show fertility rates remain higher for Aboriginal and Torres Strait Islander women than for the total population, [25] empirical research suggests the contrary. An audit of medical records in one Aboriginal community in the NT reported 26 % of reproductive-aged women experienced infertility, more than twice the corresponding rate for all Australian women [17]. This concurs with the observations from HCPs in this study. Providers believed that a high fertility rate likely coexists with a high infertility rate in the Aboriginal and Torres Strait Islander population.

In low-income country settings, high rates of infertility are often the sequelae of poorly managed or untreated STIs [7]. Similarly, HCPs in this study reported encountering many STIs and recurrent episodes of PID. Aboriginal and Torres Strait Islander people have substantially higher rates of STIs than non-Indigenous Australians, for chlamydia and gonorrhoea [26]. In the NT, after adjusting for differences in population age structure, hospitalisations for acute PID occurs at four times the rate among Aboriginal and Torres Strait Islander women, than for non-Indigenous women [27]. Interestingly, PCOS, which is the most common cause of anovulatory infertility, and also more prevalent among in Aboriginal and Torres Strait Islander women, [28, 29] was not mentioned by the health practitioners in this study. This may be because PCOS is often misdiagnosed or underdiagnosed [30]. The degree to which PCOS impacts on fertility in this population requires further research.

The importance of effective patient-provider communication was strongly represented in this study. Effective communication is not only critical for accurate diagnosis and providing patient-centred care, it is also highly correlated with better patient adherence [31, 33, 34]. Considering the time-sensitive and user dependant nature of fertility treatments, patient-provider communication is of particular importance. Many factors can influence the effectiveness of communication. In our study, providers spoke of language barriers, low health literacy and cultural differences. Similar findings have been reported in other healthcare settings caring for Aboriginal and Torres Strait Islander clients [32–34]. Effective communication is a ‘two-way’ process. In the Aboriginal and Torres Strait Islander context, ‘two-way’ communication involves bringing together Aboriginal and Torres Strait Islander and non-Indigenous knowledge, world-views and process, and giving them equal status [35]. Central to this is a bi-directional dialogue, which emphasises careful listening by HCPs [36]. Given most Aboriginal and Torres Strait Islander people in the NT primarily speak an Indigenous language, [37] this often relies on the services of Indigenous language interpreters [38]. However, when discussing challenges in cross-cultural communication, very few providers in this study mentioned using qualified Indigenous interpreters, despite most Aboriginal and Torres Strait Islander clients being eligible for interpreting services through Commonwealth funding. This may reflect problems of availability, accessibility and quality of interpreter services, or a lack of awareness of interpreter services by HCPs. Providers should promote the engagement of interpreters when required and look to adopt other strategies shown to improve communication practices in Aboriginal and Torres Strait Islander health care such as diagrams, illustrations and culturally meaningful analogies [33, 39].

Providers’ perceptions of stigma associated with sexual health and the subsequent personal shame was commonly discussed. Providers felt these attitudes were more pronounced in men, evidenced by very few encounters of men presenting with infertility concerns. Indeed, others have highlighted Aboriginal and Torres Strait Islander people’s concerns about the stigma that can be associated with culturally sensitive health issues such as sexual and reproductive health and have shown this stigma to impact related care seeking [40–42]. Further research is needed to confirm whether infertility is considered a sensitive issue and experienced as stigmatizing in this population. This research should be conducted with the utmost care and involve Aboriginal and Torres Strait Islander peoples from the outset to ensure that work done in this space is culturally appropriate, respectful and meaningful.

Providers described several structural and service-related barriers. High out-of-pocket costs for patients was a concern among HCPs. This is consistent with findings from a range of international studies, as while the cost of IVF treatment is variable among countries, it is generally an expensive treatment [43–45]. In Australia, navigating infertility with IVF typically costs between $9,000-$15,000 (per cycle), with out of pocket costs^6^ of around $4500 [12, 13]. These numbers do not include necessary IVF-related medications and associated hospital/day surgery related services e.g. egg pick up and embryo transfer. Private health insurance cover can further reduce some of the out of pocket costs, however not all insurance policies cover IVF treatments, and those do those that do may vary in terms of which services are covered and to what extent [46]. Statistics show that Aboriginal and Torres Strait Islander people, particularly those living in rural and remote arears, are less likely to have private health insurance than other Australians [47, 48]. To reduce out of pocket costs, some services offer bulk-billed IVF services, lower cost treatment strategies and discounts to concession card holders. However, it is unclear how or if these factors make ART services any more affordable for some patients. More generous public financing, either through insurance reimbursement, government-sponsored IVF clinics or an increase to Medicare rebates may help make services more available to low-income individuals and couples.

Other related barriers discussed by providers included a lack of modern facilities and equipment needed for infertility investigations, limited availability of local infertility specialists and distance to fertility related healthcare, particularly for rural and remote communities. Where rural patients are required to travel long distances to access specialist services, including fertility services, the PATS provides subsidies towards the cost of travel and accommodation. According to the PATS guidelines, patients receiving treatment for infertility are eligible only where there is a diagnosis of clinical primary infertility [49]. In this study, HCPs reported an impression that primary infertility was less common than secondary infertility among Aboriginal populations. If this is the case, many of patients would be disqualified from access to the PATS, potentially making travel unaffordable. Attempts could be made to tailor the travel scheme to better assist Aboriginal and Torres Strait Islander patients from rural and remote areas who need to travel to access fertility care.

While improving patient access was a priority for providers in this study, many felt powerless and poorly equipped to initiate sustainable change. With an already overstretched rural health system, [50] there were concerns that advocacy for infertility would divert resources from patients with chronic diseases, and therefore exacerbate health inequities. Others viewed infertility as a reproductive rights issue and therefore considered advocacy for fertility treatments as part of protecting those rights. The ethics of infertility treatment is typically discussed in the context of low-resource countries e.g. Africa [51, 52]. Whilst the barriers to provision of ART in low-resource countries are continually cited, the importance of a person’s reproductive autonomy, as emphasized by the World Health Organisation, demands that efforts should be made to ensure people have the right to decide when, how many and how to have to children [53]. Widely advocated strategies to improve accessibility of fertility treatment in low-resource countries include the development of low cost and simplified ART procedures. To date, the effectiveness of low-cost technology is encouraging [6]. However, against the ongoing impacts of colonisation on the health and healthcare of Aboriginal and Torres Strait Islander people, this discussion is highly complex, and goes beyond the influence of socio-economic circumstances. More studies are needed to assess whether these strategies can be replicated in other settings such as Australia, and the efficacy and feasibility in the Aboriginal and Torres Strait Islander context.

Generally, providers felt they played a role in patient education. This was mostly described in relation to promoting fertility awareness, healthy behaviour change and reproductive life planning. Emphasis was placed on the importance of this being culturally sensitive, beginning in youth and continuing into adulthood [54]. Providers felt that fertility services have a responsibility to provide culturally appropriate care and support. This includes ensuring environments are inviting, respectful of culture and increasing the number Aboriginal people and male and female staff. Culturally safe interventions have a positive effect on patient satisfaction, confidence in health professionals, and health service access and use, [55, 56] and a “trend in the direction of a positive impact” on patient outcomes [57]. Finally, several providers suggested that services should focus on providing regular clinical workplace education sessions or useful resources to strengthen provider knowledge around fertility treatment options, particularly those working in primary care where cases of infertility care are infrequent. Today, clinical practice guidelines are an important component of medical education and ongoing training process. There is currently no Australian consensus guidelines for the diagnosis and treatment of infertility. Developing new guidelines for infertility should be undertaken as a priority to promote best-practice evidence-based infertility care and needs to include specific recommendations that address the needs of Aboriginal and Torres Strait Islander people. Against the backdrop of resourcing priorities and decisions that have created a resource-limited environment, the potential community and individual payoff of health promotion is immense.

This study adds considerably to the body of knowledge around access to and uptake of infertility services among Aboriginal and Torres Strait Islander people, by identifying the most significant barriers, as perceived by health providers, to diagnosis and treatment in this group. Our findings are reflective of attitudes and opinions held amongst healthcare providers from one region of Australia. Hence, as expected in qualitative studies, the results may not be representative of fertility care in other regions. This study is based on interview data and there may be discrepancies between the information retrieved and infertility care in the NT, and other Australian jurisdictions. Currently fertility clinics in Australia are not required to collect Aboriginal and Torres Strait Islander status as part of their mandatory reporting to ANZARD. Therefore, it is possible that subjective assumption of Indigenous status may have led to some underestimation of utilisation of infertility services by this group. Further, HCPs willingness to participate in the study may have contributed to selection bias. The study team overcame this by selecting providers from different professional backgrounds, specialities and sites. Future studies will require interviews with Aboriginal peoples themselves to better understand Aboriginal perspectives on access and uptake of fertility services.

## Conclusions

The prevalence of infertility among Aboriginal and Torres Strait Islander people is poorly understood and rates are likely to be underestimated. Our results suggest that this could be caused by a number of barriers to access to infertility care and ART for this population. Issues identified by HCPs were related to culture, healthcare systems and healthcare service. Cultural responsiveness within fertility services, including Aboriginal employment provision and gender specific staff, could improve access to these services for Aboriginal and Torres Strait Islander people. More culturally relevant infertility education (for individuals and communities), which was recognised as an important part of the HCP’s job, could improve engagement and adherence to treatment. Finally, but perhaps most importantly, in-depth qualitative research with community service users, should also be undertaken to explore community’s perception of infertility and to determine whether infertility is an important community health issue. This should include men’s perceptions of infertility and experiences of infertility and infertility treatment. If identified as a priority, future directions should include partnering with communities to guide strategies for improving access and affordability to infertility care for Aboriginal and Torres Strait Islander people.

## Supplementary information

**Additional file 1.**

## Data Availability

The datasets generated and/or analysed during the current study are not publicly available due the privacy of participants but are available from the corresponding author on reasonable request.

## Footnotes

1 A Clinical Quality Registry comprising information on all ART treatment cycles undertaken in Australian and New Zealand fertility clinics.

2 Traditionally Aboriginal and Torres Strait Islander people maintained distinct gendered realities. Many communities continue to observe “men’s business” and “women’s” business, both in traditional law (lore) and cultural practices and in relation to health and wellbeing.

3 There is currently no consistent definition of Aboriginal and Torres Strait Islander Health Workers and Health Practitioners across Australia. In the NT, ‘Aboriginal and/or Torres Strait Islander Health Worker’ is term to collectively describe an Aboriginal and/or Torres Strait Islander person who is in possession of a minimum qualification (cert III) within the fields of Indigenous Community Health and/or Aboriginal Health Work. In some instances, current employment in the healthcare sector will alternatively satisfy. These roles are non-clinical. Alternatively, an ‘Aboriginal Health Practitioner’ is a person who is in possession of a minimum Certificate IV in Aboriginal and Torres Strait Islander Health Practice or equivalent and is also registered by the Aboriginal and Torres Strait islander Practice Board. These roles are clinical.

4 Patient Assisted Travel Scheme (PATS) is a subsidy program that provides financial help for travel and accommodation expenses to  patients (and eligble escourts) in remote Australia who need to travel a long distance to see an approved medical specialist.

5 To qualify for the subsidy the patient needs to have a valid Health Care Card (both patients if a couple).

6 The difference between doctor/clinic fee and the standard Medicare rebate.
