# Supplementary material for: “We are only looking at the tip of the iceberg in infertility”: perspectives of health providers about fertility issues and management among Aboriginal and Torres Strait Islander people
Source: BMC Health Serv Res. 2021 Jul 17;21:704. doi: 10.1186/s12913-021-06714-8 (PMC8285865; doi:10.1186/s12913-021-06714-8)
Supplement: Supplementary file 1 — Additional file 1. [file 12913_2021_6714_MOESM1_ESM.docx]

**“We are only looking at the tip of the iceberg in infertility”: Perspectives of health providers about fertility issues and management among Aboriginal and Torres Strait Islander people.**

**Interview Guide**

This interview guide provides a brief overview of the project ‘A qualitative exploration of health care provider’ perspectives on infertility among Aboriginal and Torres Strait Islander (Indigenous) People in the Northern Territory” as well as an explanatory note to ensure participants are able to give informed consent. Participants will be asked if they have any questions and if they agree to the audio recording of the interview before being asked to sign the consent form. In the case of phone interview provide, they will be asked to give verbal consent before the interview.

***Background***

In many Indigenous communities, women and men are disproportionately affected by a range of risk factors for infertility including sexually transmitted infections, polycystic ovary syndrome and obesity. However, very little is known about the occurrence of infertility in this group, or how Indigenous people access fertility services or treatment. However, we do know that accessing services may be difficult due to cost, location and most likely culturally responsive care.

***Current project***

To improve our knowledge and understanding of Indigenous infertility, we are interviewing healthcare providers involved in infertility care and treatment. Insights from these healthcare providers will provide an opportune understanding of the current provision of fertility care to Indigenous people and will assist in identifying areas where service gaps exist. This information will be fed back to participating healthcare providers to help improve service delivery, and ideally lead to fertility improvements within the Indigenous community. This semi-structured interview consists of 10 open-ended questions focusing on three main areas: (1) prevalence of infertility among Indigenous people, (2) access and uptake of fertility services, and (3) barriers and facilitators towards fertility treatment for the Indigenous community.

Interviews are expected to take approximately 30-45 minutes each. The research team will use information gained from interviews to guide the development of a survey which will be widely distributed among health providers who work in the reproductive and fertility fields in Top End and surrounds. This two-phase research methodology merges the depth of interviews with the breadth of an online survey.

The project is part of a larger program of work that aims to understand and improve the provision of fertility services to Indigenous women and men.

**Demographic information**

Age:

Gender:

Role:

How long have you being living in the NT?

**General questions**

**Q1. How do you currently provide infertility care or treatment in your routine practice?**

**Probes:**

What is your position title?

At what stage of the fertility treatment/care process are you involved in? What sites do you work from – RDH clinics, outreach etc.

**Q2. What are the demographic characteristics of men and women seeking fertility treatment?**

**Probes**:

What age? How long have they been trying to achieve a pregnancy for?

What disparities, if any, are there regarding age, race/ethnicity, sexual preference, socio-economic status?

**Topic Area 1: Prevalence of infertility among Indigenous people**

**Q3. How much of an issue is infertility within the Indigenous community?**

**Probes:**

What proportion of the total clients requesting or accessing fertility treatments at your practice identify as Indigenous?

Do couples usually attend together?

In general, how complex are infertility issues within your Indigenous client population?

What are the major causes of infertility among your Indigenous clients or the wider Indigenous community as a whole? Example ovulation induction, tubal, male factor, reversal of tubal ligation etc.

**Topic Area 2: Indigenous access and uptake of fertility services**

**Q4. How would you describe Indigenous access and uptake of fertility services?**

**Probes:**

Do Indigenous people experience worse, same or better access to fertility services than the non-Indigenous community?

Is uptake of fertility services among Indigenous people worse, same or better than non-Indigenous people?

What are the major determinants of utilisation of fertility health services among the Indigenous community e.g. educational status, metropolitan/rural/remote living, socio-economic status,

**Q5. What challenges are there in providing technically competent fertility care for Indigenous people?**

**Probes**:

Are there issues associated with patient geographical location that impact proper delivery of care e.g. failure to attend follow-up appointments, need for consideration and integration of travel requirements, lack of basic communication infrastructure within communities (telephone, internet), lack of communication between health services, need for many tests, test needing to be done in a major centre?

Do you find adherence to medical regimes an issue amongst Indigenous patients? If yes, what are some of the factors that may affect this?

**Q6. How do the needs of Indigenous people in your community differ from non-Indigenous communities with respect to infertility care and treatment?**

**Probes:**

Are you aware of any cultural considerations surrounding health or seeking treatment for infertility within Indigenous communities?

**Topic Area 3: Barriers and facilitators towards fertility treatment for the Indigenous community**

**Q7. How well do fertility services cater for Indigenous people?**

**Probes**: “one-size fits all” approach, constructed values at odds with Indigenous communities’ beliefs and values, influence of the biomedical vs. holistic sense of health,

**Q8. What barriers, if any, exist in accessing fertility services for Indigenous people?**

**Probes:**

What, are some of the *logistical barriers* e.g. challenges associated with geographical isolation and access and uptake : problems with access to and shortage of providers, services and specialist care, costs of travel and accommodation, restrictions on escort eligibility (PATs) and access to transport, time away from community (family & work commitments), health literacy, lack of anonymity, transport of samples.

What are some of the *cultural barriers* e.g. concentration on Western health concepts, lack of Indigenous health professionals, differing language, (need for an interpreter), patient comfortability with certain diagnostic tests and treatments (e.g. bloods, ultrasound, semen analysis), societal stigma, traditional and religious beliefs, gender-based responsibilities, support from family-relationships, Gender of health care worker – female O&G taking to men, male O&G talking to women

What are some of the *systemic barriers* e.g. cost of treatments (large out of pocket treatment costs associated with ART services), private health insurance coverage, experiences of discrimination, racism and poor cultural understanding, mistrust of healthcare system.

**Q9. As a health provider, what role do you think you play in removing barriers to improve health service delivery for Indigenous people?**

**Probes:**

Are there changes to your practice which you could you introduce to work more effectively with, and improve service delivery for Indigenous clients.

**Q10. What strategies do you think services could employ to improve access to fertility care for Indigenous people?**

**Probes:**

Partnerships with local communities, bulk billing for all Indigenous patients (by all agreeable doctors), transport to fertility service (community run bus), Indigenous liaison support, provision of out-reach services and home visits, employing Indigenous staff, cultural awareness talks to staff, Indigenous spaces (flags, artwork, culturally appropriate waiting room) etc.
